# Feasibility of high dose dobutamine stress and scar imaging in high field open MRI in patients with suspected coronary artery disease

**DOI:** 10.1186/1532-429X-15-S1-P197

**Published:** 2013-01-30

**Authors:** Phillip G Petry, Dirk Lossnitzer, Sebastian M Haberkorn, Fabian aus dem Siepen, Sebastian A Seitz, Mohamed A Abdelrazek, Henning Steen

**Affiliations:** 1 Department of Cardiology, University of Heidelberg, Heidelberg, Germany; 2 Radiology, Cairo university, Faculty of Medicine, Cairo, Egypt

## Background

High-dose dobutamine-stress magnetic resonance (DSMR) and late gadolinium enhanced cardiac magnetic resonance (LGE-CMR) imaging are well established for the detection of significant coronary artery disease (CAD) and infarction for cylindrical 1.5 T or 3T. Due to the narrow bore, obese or claustrophobic patients (pts) cannot be studied with conventional MRI scanners. 1.0T high field open (HFO) is a non-cylindrical MRI system offering significantly more space but has only rarely been utilized for diagnostic cardiac MRI so far.

We sought to investigate the performance of 1.0T HFO DSMR and LGE-CMR in pts with suspected CAD and low to intermediate risk profile.

## Methods

We studied 80pts (58men;32women,59±12ys.) with low to intermediate PROCAM risk-score for significant CAD not eligible for 1.5T due to obesity (44pts) or claustrophobia (36pts) in a 1.0T HFO (Philips Panorama). Dobutamine was administered using established standard protocols, atropine was given to reach heart-rate target if necessary. We employed a standard ECG-and or pulse-gated SSFP-sequence (TR/TE:4.7/2.2msec,flip-angle:70°,resolution:1.8×2×8mm3,30 heart phases) for DSMR and a double inversion-recovery sequence (TR/TE:3.9/1.3msec,flip-angle:15°,voxel:1.7×1.9×5mm3,TFE-factor=21) for LGE-CMR. Two-, three- , four-chamber and three short axes image planes were taken step-wise at rest and stress. After stress imaging, LGE CMR was performed to detect potential small myocardial infarctions.

## Results

DSMR and LGE-CMR were successfully performed in 78/80pts. Average stress heart rate was 94±7% of age-predicted maximum heart rate. Patient-based sensitivity and specificity were 78.0% and 83% respectively and accuracy was 84%. Inter-observer variability for assessment of wall motion abnormalities was 84% (κ=0.740;p<0.043). Negative and positive predictive values were 71% and 89%, respectively. Sedation could be avoided in 85% of all claustrophobic pts. Mean body-mass-index was 29±6. In 19% of pts, the vector ECG-signal became insufficient under stress at heart rates >120/min but additional peripheral pulse-gating was sufficient in all cases.

## Conclusions

DSMR and LGE-CMR at 1.0T HFO is a feasible, accurate and sedation-saving method in obese or claustrophobic pts to enable diagnostic MR stress and scar imaging in this usually not accessible patient collective. DCMR at 1T is an accurate method to depict significant coronary artery stenosis in patients with suspected or known CAD. Due to the increasing number of obese pts, 1.0T HFO could represent a valuable alternative for conventional narrow bore CMR machines. Although vector-ECG was lost in approximately one sixth of pts, potentially due to the pronounced magneto-hydro-dynamic effect in vertical magnetic fields, pulse-gating was sufficient to terminate all DSMR and LGE-CMR scans successfully.

## Funding

none

